# BadDomains: Early Detection of Phishing Domains Registration

**DOI:** 10.3390/s26031041

**Published:** 2026-02-05

**Authors:** Karolina Seweryn, Piotr Białczak, Tomasz Chytry-Trzeciak

**Affiliations:** 1 NASK National Research Institute, Kolska 12, 01-045 Warsaw, Poland; karolina.seweryn@nask.pl; 2 CERT Polska/NASK, Kolska 12, 01-045 Warsaw, Poland; tomasz.chytry-trzeciak@cert.pl

**Keywords:** phishing detection, phishing domain, machine learning, cybersecurity, cybercrime

## Abstract

Phishing attacks often rely on impersonating a legitimate entity, such as a well-known company or a bank, with the intent to deceive individuals. A common tactic used by cybercriminals to conduct such an attack is to register a specific domain to host a phishing website on it. In this paper, we propose BadDomains, a system for the early detection of phishing domains’ registration. BadDomains utilizes domain registry data about newly registered domains combined with knowledge about the current phishing situation, such as information about the most frequent impersonation targets, or suspicious domain contact information. An analysis of *.pl* phishing domain registry data, combined with the authors’ CSIRT operational experience, helped in the design of new features. It also facilitated the extension of features already used in other solutions. The system’s evaluation has been performed using information from *.pl* Top Level Domain (TLD) registry combined with CERT Polska’s (Polish national CSIRT) public list of phishing domains, used as a ground truth. BadDomains has been compared to a similar detection system designed for *.eu* TLD called Premadoma, which was adapted to this work. The results showed that BadDomains achieved higher $F_{1}$ scores than Premadoma. After operational deployment, the system proved to provide timely detections, uncovering unknown phishing domains.

## 1. Introduction

Phishing attacks are used to trick people into providing sensitive information like login credentials or credit card information. Cybercriminals masquerade as legitimate businesses or well-known entities to gain victims’ trust and persuade them to reveal sensitive information. Phishing is one of the main threats to today’s internet [1]. Unfortunately, it is still on the rise [2], making it the main cyberattack targeting the general public in some countries, e.g., Poland [3].

Various approaches address this problem using blacklist/whitelist mechanisms, textual or visual similarity analysis, and machine learning models (cf. R. Zieni et al. [4]). The methods analyze various artifacts, such as Domain Name System (DNS) network traffic, passive DNS data, domain names, Uniform Resource Locators (URLs), domain registry data, or website content (cf. A. Khormali et al. [5]). However, many of these methods operate using data obtained during or even after deployment of the attack, when many victims have already been lured to share sensitive information.

To address this issue, we propose BadDomains, a system for the early detection of phishing domains’ registration. The system uses data from the Top Level Domain (TLD) registry about recently registered domains in order to predict which domains are likely to be used for phishing in order to try to react to them before they are used by criminals. A domain registry contains data about domains (e.g., their names, times of registration), entities facilitating registration of domains (registrars), and domain owners’ (registrants) contact information. A set of features is extracted from the data and input to a machine learning model based on LightGBM [6]. Additionally, the data is enriched with information from additional services such as a geographic map service or IP address geolocation. Leveraging our operational experience handling phishing incidents at Poland’s national CSIRT (CERT Polska), we have designed innovative features not previously proposed. These include the presence of trademarks of the most frequently impersonated targets in domain names and the identification of domain contact information that has been used in past phishing attacks. These features are integral to a key system component that utilizes up-to-date knowledge about the current phishing situation. This information is continuously collected and updated through the handling of phishing incidents. BadDomains uses this data for enrichment and feature extraction purposes. The data includes information about the main targets of phishing impersonation and strings frequently used in phishing domains.

BadDomains has been designed to operate using data from the *.pl* TLD registry. For training and evaluation, CERT Polska’s public list of phishing domains targeting Polish internet users [7] was used as the ground truth. The system has been compared to a similar detection system designed for the *.eu* TLD called Premadoma [8] and to a baseline system analyzing only domain names and checking for the presence of strings frequently used in phishing domains. Premadoma was chosen based on its similarity to our approach to detecting domains early after their registration, usage of similar features and data, and technical soundness. These three approaches have been compared using standard classification metrics: precision, recall, and $F_{1}$. The systems were evaluated on a data set that covers domains registered in the *.pl* TLD between 1 January 2023 and 31 March 2024 (15 months). Furthermore, BadDomains was deployed in CERT.PL’s phishing detection environment. As this is a real-world operational scenario, the system was further evaluated during the deployment period between 29 November 2024 and 28 July 2025 (8 months).

Our work is intended to address problems encountered in a threat model in which cybercriminals utilize a TLD to perform multiple phishing attacks. In such a threat model, the attacker can be a single person or multiple, independent, organized criminal groups that register domain names as single domains or in sets. The defender is a TLD registry securing its domain zone or a national-level CSIRT team fighting cybercrime in its constituency. Optionally, they could be a CSIRT responsible for a TLD in the case of TLDs other than country code ones. The defender can block a domain name at the registry level (in the case of a TLD registry), report it to a registry, or add a domain to a blocklist, which can be used by responsible entities to block network traffic to the malicious domain.

Considering the above, the main contributions of this paper are

- proposing a new method for the early detection of phishing domains using domain registry data and knowledge about current phishing campaigns,
- designing new model features based on operational experience gained during the handling of phishing incidents and extending features proposed by other researchers (in total, we implemented 77 novel or partly novel features out of 224 used by our model),
- analysis of phishing domains in the *.pl* TLD using domain registry and incident report data, with application of obtained knowledge to design a detection system,
- evaluation of the system during deployment in a real-world operational scenario of working as part of CERT.PL’s phishing detection environment,
- demonstrating that the proposed system identifies phishing domains absent from existing CERT.PL ground-truth data, revealing false negatives and providing a mechanism for improving detection coverage and ground truth quality.

To the best of our knowledge, this approach is the first phishing domain detection system that integrates domain registry data with insights from current phishing activities collected by experts during phishing incident handling.

## 2. Related Work

As stated in the Introduction, many different phishing detection techniques exist, using various artifacts. For example, some recent works discuss the problem of using domain names for the detection of fraudulent websites. In [9], F. Deniz et al. present a system for the detection of phishing domains by using the hosting and lexical features of domains for the creation of a Graph Neural Network classifier to uncover domains unknown to some popular domain blacklists. M. Bitaab et al. in [10] proposed a system for the detection of fraudulent shopping websites. They utilize a feed of daily domain registrations as input to their detection pipeline and perform an extensive analysis by collecting domain information, classifying domains as to whether they are potentially fraudulent shopping websites, and finally collecting merchant IDs from payment processors. Y. Nosyk et al. in [11] have chosen a different research approach and analyzed what factors cause the usage of particular Top Level Domains and registrars. They also analyzed what features are linked to malicious domain registrations.

However, in order to better present our results, we will focus on a review of work directly addressing the detection of newly registered phishing domains. This approach provides a way to react to the threat before it can be widely used by attackers. One way to achieve this is to monitor DNS network traffic in order to identify requests for newly created domains. A recent example of such a method is [12] by Silveira et al., in which the authors propose a malicious domain detection system based on passive DNS data collected from an authoritative TLD server. A set of features is extracted and fed to a LightGBM-based model.

Another approach focuses solely on the domain information available, for example, at the registry level. This approach is used in our work, and it has already been implemented by other researchers. For example, in [13], authors proposed a malicious domain prediction system called Predator. They use Convex Polytope Machine algorithm with a set of features based on groups such as domain profile, registration history, and batch registration correlation. The first group consists of features modeling the domain, like registrar, time of registration, and presence of particular character types. Registration history consists of features connected with the previous registration of the domain. Finally, batch registration correlation groups features that model relations between domains registered in batches. In [14], the authors focused on phishing detection using solely Turkish domain names. They designed a convolutional neural network (CNN) and applied character-level embeddings to create a detector. In [8], the authors propose a malicious domain detector called Premadoma. They utilize a set of features based on a domain’s registration information, its name, registrar information, and registrant contact information, including features that model reputation, that is, the previous history of usage in malicious activity. A majority voting architecture is used, consisting of two reputation-based classifiers and a similarity-based clustering module. More information about this system can be found in Section 4.2. Another approach was presented by Y. Tian et al. in [15]. In the paper, the authors create a malicious domain detector called Dom-BERT. They create graphs that model relationships between domains, clients, and hosting servers. The graphs are used to compute similarity scores for domains, which are then used to fine-tune the pre-trained BERT model. F. Çolhak et al. in [16] proposed a method for malicious domain name registration detection which operates solely on the domain name but combines two approaches for data analysis. In the first part, the system extracts features from a domain name, such as string length or number of digits, and adds information about the similarity score to already registered domains. The features are then fed into a multilayer perceptron. The second approach creates an embedding of a domain name using a transformer architecture. The output of those two approaches is combined into a linear classifier. In [17], T. Daniels et al. presented RegCheck, a system for the detection of potentially malicious *.be* domain names, including phishing. As their system flags domains at the moment of registration to be verified manually, the authors put limitations on the number of domains that could be verified. Thus, their system maximizes the recall of detection but still limits the number of detected domains in a given 7-day window. The authors described two mechanisms for decision threshold selection and results for various learning algorithms, including Random Forest and LightGBM. Model features included reputation, geographical information, domain lifecycle, and lexicographic information, including TF-IDF on character n-grams of contact information and domain name. In [18], A. Del Soldato et al. described READS, a system for anomaly detection in the registrant data of *.it* domain names. Domains detected by the system as having some information inconsistencies or other anomalies are then forwarded for manual correction. The authors presented two detectors, one supervised, using an LSTM architecture, and one unsupervised, using an autoencoder architecture. Both use the same feature set based on registrant information such as the name of the registrant, their address, nationality, and registration code, such as fiscal identifier or VAT identifier.

In our opinion, the work presented above has some weaknesses. The method of Silveira et al. [12], despite focusing on newly registered domains, has a delay in providing prediction, thus potentially making the reaction time of the defenders shorter. The Predator and Premadoma detection systems use domain registry data but lack deeper analysis of data consistency and validity. Also, usage of clustering in Premadoma can potentially be helpful for tracking broader campaigns involving many domains and a few registrants, but it can be a hindrance in the detection of one-shot phishing attacks. READS focuses on anomaly detection in registrant information but not on phishing detection per se. As the authors did not analyze it directly, it cannot be determined without additional analyses whether it would be efficient in phishing detection, as cybercriminals could provide non-suspicious registrant information. RegCheck, similarly to READS, focuses on providing a set of domains for additional verification in order to decrease the number of malicious domain registrations. As such, it is not focused on phishing detection, and its objective function of limiting the number of detected domains can compromise both precision and recall. Based on the model feature description, the system does not use any operational information, which can hinder the tracking of current threats. Systems focusing only on domain name, like Dom-BERT, the method proposed by F. Çolhak et al. in [16], or the method proposed by Bozogullarindan et al. in [14], are strongly limited by primarily or only using the name, what can prevent the system from finding connections in data, thus limiting prediction capabilities. Moreover, none of the above methods utilizes operational information from handling phishing incidents. BadDomains addresses these issues by using all available domain registry information, and enriching it with external data, within a short time after domain registration. By using operational information about the current phishing situation, it additionally supports the prediction process with up-to-date data.

## 3. Proposed Method

The following section describes the proposed phishing domain detection method. First, we present the system architecture, followed by the features used by the machine learning model.

### 3.1. System’s Architecture

**Figure 1:**
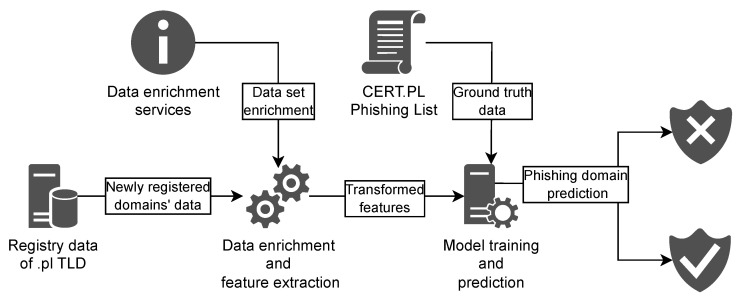
Architecture of the proposed method.

The proposed method’s architecture is presented in Figure 1. The system is based on data from newly registered *.pl* domains. This data is collected over a defined period and stored to form an analysis dataset, which is then split into training, validation (hyperparameter selection), and evaluation subsets, as presented in Section 4.1.

The data is enriched using several services, such as a locally deployed geographic mapping service based on Nominatim (https://nominatim.openstreetmap.org/ui/search.html, accessed on 27 November 2025), IP address geolocation, IP address to Autonomous System Number (ASN) assignment, and telephone and e-mail reputation services. All services operate entirely within the internal infrastructure, ensuring that no data is transmitted outside the organization. Using the enriched data, feature extraction is performed in order to transform the data into defined features, which are fed into the machine learning (ML) algorithm.

For modeling, we use LightGBM [6], a gradient-boosting framework based on decision trees. It is distinguished by its speed [19], efficiency, and predictive effectiveness, making it a popular choice in numerous projects. During model training and evaluation, we use *.pl* domains from CERT Polska’s public Domain Phishing List [7] as the ground truth. Once trained, the model is applied to the stream of newly registered domains to identify potential phishing domains.

### 3.2. Model’s Features

The system’s input data consists of information stored in the DNS registry. These include registrants’ (domain owners’) contact information (name, email, postal address, telephone), information about the registrar (the entity registering the domain at the registry on behalf of the registrant), timestamps of registration and updates, or the domains names’ server information. Most of the data is stored as strings and is therefore not suitable for direct machine learning model training. Therefore, we performed feature engineering, which was a critical phase of our study. The designed features can be divided into 8 groups:

- Basic features, which are extracted directly from the input data, e.g., country code of a registrant, identification of the registry, or the domain name’s server’s IP address version.
- Contact validation, which consists of features that check contact information for any inconsistencies, syntactically validate some fields (e.g., phone number), model the occurrence of values, or determine if the respective contact fields are empty. For example, geolocation data from Nominatim was checked for coherence with the information provided by the registrant. We have developed a scale to measure address correctness that reaches its maximum value when the entire address is accurate. Intermediate values are given when certain elements, such as the postal code, are incorrect.
- Nameserver-based, which models information about a domain’s authoritative name server that includes its IP address’ respective Autonomous System Number (ASN), its geolocation, or information about the service company.
- Keyword-based, that model if a domain contains words from a curated list of keywords maintained by CERT.PL’s phishing incident handlers and automatic systems. The list represents the current threat situation by covering abused trademarks, words used by cybercriminals in other phishing domain names, and other words of interest. Besides the presence of specific keywords within the domain, Damerau and Jaro edit distances are calculated for the domain name and the keyword list.
- Lexical, which extracts information from textual fields such as domain name, registrant’s name, e-mail address, and postal address. The features extract data such as the percentage of vowels, string length, unique symbol count, digits, letters, and entropy levels. Also, the presence of consecutive characters is checked to identify random characters pressed on a keyboard.
- Reputation-based, in which features model the prevalence of phishing domain registrations per register, nameserver service provider, or e-mail provider. Additionally, we used information on whether a phone number or an email address had already been used to register phishing domains, with a special case for the last 90 days. An additional feature was created for phone numbers: whether they are listed on a separate list of suspicious numbers created by phishing incident handlers and automated systems.
- Similarity-based, where features model the Levenshtein similarity of a domain name to previously registered domains and previous phishing domains. Additionally, Levenshtein and Jaro edit distances are calculated for the company names most targeted by phishing.
- Time-based, which extracts information about the domain creation date (including hour and day of the week) and time distances between previous and next registrations. Considering the potential relevance of certain aspects from prior registrations, we calculated the number of domains registered in the last minutes.

A detailed description of the full feature set is omitted from the text for the system’s operational security, as the system operates in an adversarial environment involving cybercriminals. However, the complete specifications with architecture details can be provided to bona fide researchers upon request. In total, we created 224 features for the model, of which 77 are novel or partly novel. These are presented in aggregated form in Table 1. Table 2 presents the remaining model features, namely those previously proposed by other researchers and not novel.

## 4. Experimental Evaluation

In this section, we describe the experimental method used to assess the performance of BadDomains.

### 4.1. Data

The input dataset consists of 942,605 observations of registered domains with registration dates from 1 July 2023 to 31 March 2024. Cases in which a known phishing domain had been re-registered were removed from the dataset. This modification was implemented because the domain may still be malicious, even though it is currently not listed as phishing in the data. The final dataset includes 5678 negative (phishing) observations, although their number varies across individual months.

**Figure 2:**
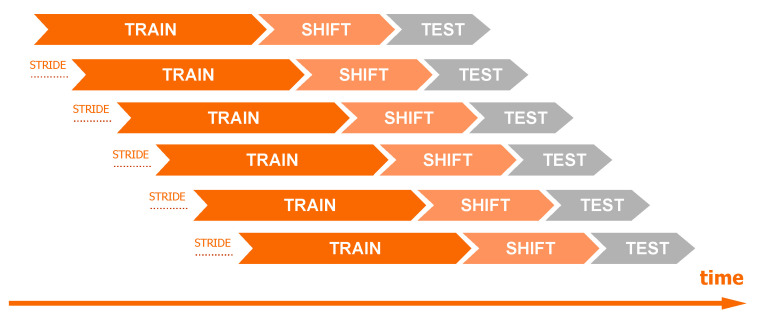
Diagram of the data split of time series using a moving window technique.

**Figure 3:**
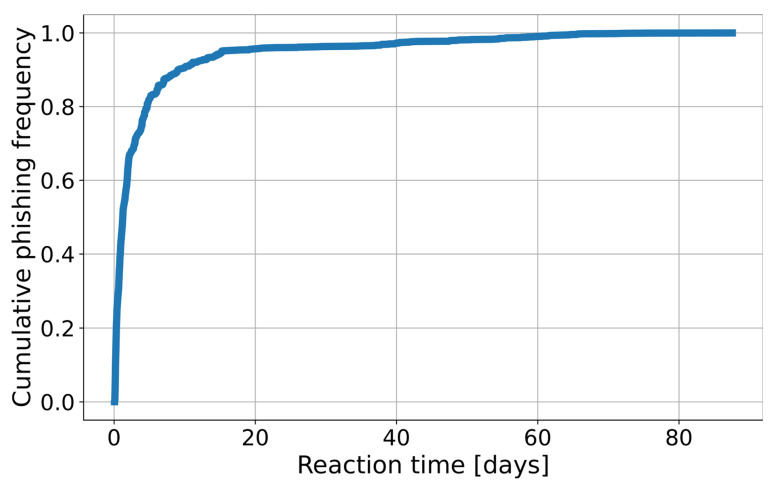
Empirical cumulative distribution function of reaction time (time between the registration and blocking of a phishing domain).

The dataset-splitting scheme is presented in Figure 2. Observations from the N days (the number of days is a hyperparameter) preceding the start date of the data shift period were used for training. A 5-day gap was used between observations from the training and test sets to simulate real conditions shift. In phishing detection, it typically takes about 5 days from the domain registration to its detection (5 days cover over 80% of phishing, see Figure 3). Consequently, using the most recent 5 days for training would risk mislabeling domains as “benign” that later prove to be phishing. We also introduced a minor modification to the training dataset: If in the observations from the 5-day gap, the phishing label was before the first day of the test dataset, then those phishing observations were added to the training. This approach ensures that the most up-to-date information on attacks is available to the model, but it does not introduce false negative observations. The “stride” variable specifies the frequency at which the model is trained. For example, setting the stride to 1 day means that data is split daily according to this scheme, and models are retrained accordingly.

In production conditions, the model is retrained once a day on the latest data, and predictions are made every two hours. This automated workflow significantly reduces reaction time compared to the detection latency observed without an automated system, as illustrated in Figure 3. For testing purposes, we used a 1-day test period with a 1-day stride between each test. This means that for a 30-day analysis, we constructed 30 distinct models corresponding to each day. However, to reduce the number of calculations during hyperparameter optimization, the approach was slightly modified. The validation set for hyperparameter optimization was divided into weekly intervals (instead of daily). Consequently, our testing and stride period for this phase is extended to 1 week. Details can be found in Table 3. The validation dataset spans a period of two months, specifically July and August. The subsequent seven months have been designated as the test set.

Notably, the dataset is highly imbalanced—the percentage of malicious domains is smaller than 1%. This is due to the fact that the system is designed to detect only phishing domains, without considering other forms of cyberattacks. Such a low percentage of malicious domains may affect the difficulty of building the model. Therefore, the BadDomains architecture includes the option to apply the undersampling technique (see Section 4.5.1.

### 4.2. Premadoma

To compare the results of our method, we have implemented a second system derived from Premadoma—a system developed to detect malicious domains at registration time in *.eu* TLD [8]. The concept behind Premadoma involves using two different algorithms—a classification algorithm and a clustering algorithm—to create three models, which are then used to give a final prediction through the majority voting rule. During the validation phase, the researchers have concluded that the best set of models is two models based on classification (each with a different set of hyperparameters) and one on clustering. Algorithms used in the original Premadoma are the PART algorithm [20] for classification and an agglomerative clustering algorithm for clustering.

Since the goal of our system differs from Premadoma’s—Premadoma was developed to detect any malicious domain, while we focus solely on phishing domains—we had to adapt it for our purposes. First, we have removed features that targeted other types of abuse and proved to be irrelevant in our dataset (i.e., features based on hex patterns, which are much more common in botnet domains than in phishing ones). Furthermore, instead of using the PART algorithm, we have used the hierarchical shrinkage algorithm [21], which is also a tree-based algorithm and acquired better results on our dataset. The difference between the original approach and ours creates an additional challenge: Classes in our dataset are much more imbalanced. In the dataset used for the original Premadoma, 2.53% of registrations were marked as malicious, which is substantially more than in our data.

Every model in our system was trained with regard to three hyperparameters for each algorithm—distribution spread, blacklist incompleteness and training window for the classification algorithm and distance threshold, minimum cluster size and training window for the clustering algorithm (as proposed in Premadoma). Distribution spread represents the proportion of phishing domains in the training dataset; due to a substantial imbalance in our data, we have been performing oversampling to achieve the given ratio. The second hyperparameter (blacklist incompleteness) was designed to minimize the noise generated by domains registered with malicious intent but never blocked in the registry; if any of the reputations of a benign domain, calculated for registrars, nameservers, email providers and phone number, was worse than the given threshold, it was omitted in the training data. The training window is the only common hyperparameter between classifier and clustering algorithms, and it represents the number of days used for model training. The distance threshold, used in the clustering algorithm, represents the maximum distance between two registrations to put them in one cluster. The last hyperparameter defined a minimum size of a cluster that would be used for making predictions about new registrations.

The main differences between Premadoma and BadDomains are summarized in Table 4.

The methods use different machine learning architectures and, consequently, different machine learning algorithms. While Premadoma focuses on the detection and grouping of malicious campaign domain names, BadDomains attempts to integrate operational knowledge into the detection process. Thus, BadDomains operationalizes intelligence from various sources not used by Premadoma, including information curated by incident handlers, in a more extensive way. Furthermore, BadDomains utilizes a broader set of features, with novel features in registrant validation-, keyword-, reputation-, time-, and similarity-based groups. Additionally, BadDomains models domain name similarity features more extensively in addition to the validation and consistency of data provided by registrants.

### 4.3. Base Model

Additionally, we have chosen a simple approach as a baseline for models’ comparison. We have compiled a list of phishing target names or words frequently used by criminals in phishing domains. The list was based on historical phishing campaigns observed to use the .pl TLD, and operational knowledge of CERT.PL security incident handlers. The analyzed domains were searched for the presence of words from the list. If at least one such word was found, the domain was marked as being used for phishing. This approach can potentially lead to a high number of false positives, e.g., some names being a part of some longer, benign words, or usage of words which are popular, such as *bank*. However, it is simple to implement, maintain, and interpret. Thus, despite its drawbacks, we have decided to add it to our analysis.

### 4.4. Classification Measures

For evaluation purposes, we use three standard measures of binary classification quality: precision, recall, and $F_{1}$. In this section, we define *TP* as True Positive (phishing domains correctly detected by the classifier), *FP* as False Positive (benign domains incorrectly detected by the classifier as phishing ones), and *FN* as False Negative (phishing domains incorrectly detected by the classifier as benign ones).

Precision is defined by Equation (1), and can be interpreted as how often a classifier does not label negative samples as positive. In the context of this work, precision can be seen as a measure of how many domains are really phishing in the set of all domains predicted by the classifier as phishing.

(1) $$\mathrm{Precision}=\frac{TP}{TP+FP}$$

Recall is defined by Equation (2), and can be interpreted as how many positive samples a classifier can find. In the context of this work, recall can be seen as a measure of how many phishing domains are properly detected by the classifier from the set of all phishing domains.

(2) $$\mathrm{Recall}=\frac{TP}{TP+FN}$$

$F_{1}$ score is a harmonic mean of precision and recall and is defined by Equation (3).

(3) $$F_{1}=2\cdot \frac{Precision\cdot Recall}{Precision+Recall}=\frac{2\cdot TP}{2\cdot TP+FP+FN}$$

### 4.5. Hyperparameters Optimization

The hyperparameters of the two models, BadDomains and Premadoma, were optimized using two different approaches as described below.

#### 4.5.1. BadDomains

In our study, we leverage the LightGBM [6] model, known for its effectiveness in handling large-scale data with efficiency and speed. LightGBM has many hyperparameters that significantly influence the model’s overall performance. These parameters, ranging from the depth of a tree to the learning rate and regularization terms, require meticulous tuning to strike an optimal balance between bias and variance, thereby preventing overfitting or underfitting. In our experiments we optimized LightGBM hyperparameters including *max_depth* (maximum depth of each decision tree, controlling model complexity), *n_estimators* (number of boosting trees in the ensemble), *learning_rate* (boosting learning rate), *colsample_bytree*(fraction of features randomly sampled for each tree), *reg_alpha* (L1 regularization term on leaf weights), *min_child_samples* (minimum number of samples required in a leaf node), and *is_unbalance* (flag to handle class imbalance by automatically adjusting class weights). Furthermore, we extended this process to the selection of other parameters such as the training window (*n_days_train*), which is a critical factor in time-series forecasting and dynamic scenarios, ensuring that our model remains adaptive and robust across different temporal contexts. A shorter time window in training can help identify the most recent patterns, but it may also turn out that malicious events from the past improve the model’s training. Therefore, we added a boolean parameter indicating whether to expand the training data with phishing observations from the past. Additionally, due to the high imbalance of data, we introduced a boolean parameter *undersampling* indicating whether to apply undersampling, and if so, in what proportion (*sampling_strategy*). In addition to undersampling, we also considered alternative imbalance handling strategies, including synthetic sample generation (SMOTE). These approaches were not selected in the final model, as preliminary experiments indicated no performance improvement. In order to choose optimal hyperparameters, we used a neural network intelligence (nni) package version 2.10 [22]. Table 5 presents the search space during hyperparameter optimization and the chosen values.

During the hyperparameter optimization process, the objective was to maximize $F_{1}$. We set the maximum experiment duration time at 2 days. A total of 2658 experiments were carried out. Table 5 presents the optimal values of the analyzed hyperparameters for maximizing $F_{1}$.

#### 4.5.2. Premadoma

Due to a relatively small number of combinations, we have decided to perform a grid search to find the optimal sets of hyperparameters to maximize $F_{1}$ score. For each hyperparameter, we have prepared a list of possible values, which are presented in Table 6. First, we have tested both algorithms separately and calculated $F_{1}$ scores for every possible set of hyperparameters for every batch in the validation set. The results thus obtained were then grouped by hyperparameter sets and averaged. Second, we have selected the best 30 sets for both algorithms and used them to find the best setup for the majority voting algorithm. As the classification algorithm achieved higher $F_{1}$ scores, we have decided to use two models based on the classification algorithm and one on the clustering algorithm (the same configuration as in the original Premadoma). For every possible combination of hyperparameters, we calculated $F_{1}$ score and chose the best one to use on the test dataset. The best combination is presented in Table 6.

### 4.6. Results

Table 7 shows the results of the analyzed models on the test dataset. For each day, metrics were calculated and then averaged, and we noted the standard deviation. High standard deviations indicate large fluctuations in daily results and are strongly linked to the small number of positive observations per day. Hence, even a single difference in prediction significantly impacts the result. As for the global $F_{1}$ score for the models (calculated on all data without averaging), it amounts to 0.79, 0.42, and 0.04, respectively, for our model, Premadoma, and Base Model.

**Figure 4:**
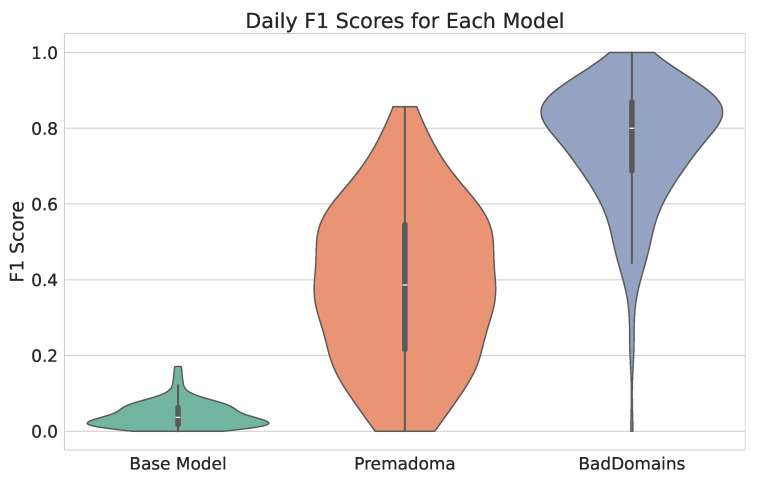
Violin plots illustrating the distribution of $F_{1}$ scores of phishing detectors.

Detection results of BadDomains significantly surpassed the results of the Base Model and Premadoma. In terms of performance metrics, the $F_{1}$ score nearly doubled, while both precision and recall increased by more than 100% compared to the Premadoma model. These relationships are also visible in Figure 4, where each violin represents the kernel density estimation of $F_{1}$ for each model. The plot highlights variations in metric distributions and central tendencies, providing a visual comparison of models’ performance. While the quality of the keyword-based Base Model remains near zero, machine learning models (including Premadoma and our solution) far surpass this basic approach. Additionally, a substantial portion of the probability mass of our model centers around the mean, whereas the Premadoma model exhibits a much wider dispersion of results. The plot distinctly demonstrates that the $F_{1}$ score distribution of our method is significantly closer to 1 compared to the competing methods, indicating the superior quality of our solution.

**Figure 5:**
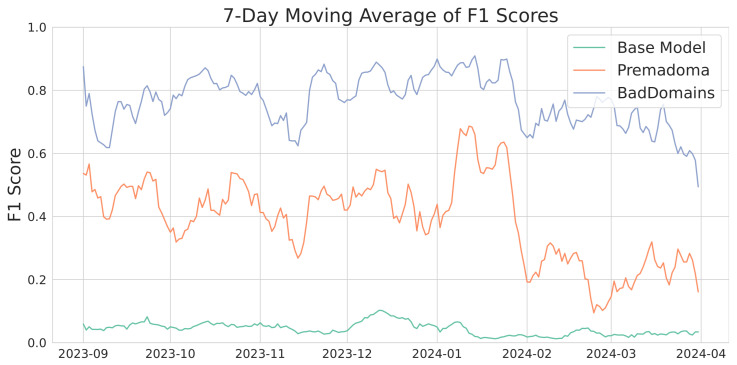
$F_{1}$ score moving average of various models.

Figure 5 shows the $F_{1}$ score metrics over time, presented as a 7-day moving average. In the latter days of March, a noticeable decline in the metric occurs, potentially due to data saturation since the observations were taken on 14 April 2024, and some domains might not have been marked as phishing despite later appearing on the list. Throughout the analyzed period, our model significantly outperforms the baselines. Interestingly, similar patterns are observed in both our model and the Premadoma model. For instance, a decrease in model quality following a previous peak in mid-January is evident in both cases. This may be caused by the use of partially similar variables and phishing campaigns, which are harder to detect during a specific period. This will be further discussed in Section 5. The Base Model is significantly worse than other solutions.

## 5. System Deployment

**Figure 6:**
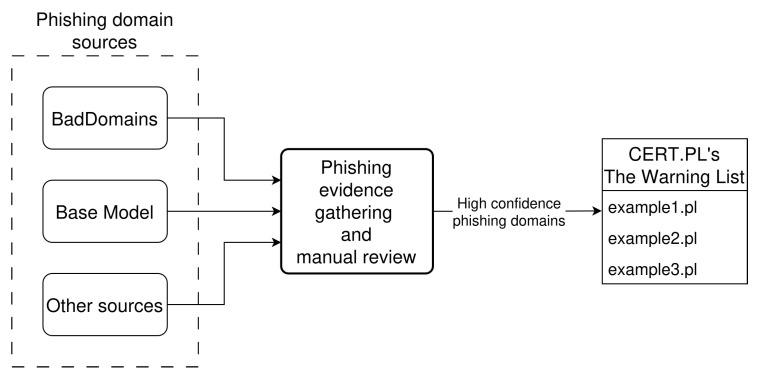
BadDomains deployment architecture in the CERT.PL’s phishing detection environment.

BadDomains has been deployed on 29 November 2024. The model operates on a server with 64 GB of RAM and 20 CPU cores. BadDomains has been integrated into CERT.PL’s phishing detection environment. The environment gathers phishing domains from various sources, collects evidence of phishing payload hosted on analyzed domains by visiting them, and a manual review of visited websites is performed. All domains identified as phishing by the detection environment are inserted into CERT.PL’s Warning List, which is also the ground truth data source for BadDomains. Besides BadDomains, only one of the sources providing phishing domains is tailored for .pl TLD, and it works on a similar principle as the Base Model presented earlier in this paper; thus, it will be treated hereafter as an equivalent of it. Other sources contain security incidents reported to CERT.PL, but also other internal systems that are out of the scope of this research. Figure 6 presents the BadDomains deployment architecture in the CERT.PL’s phishing detection environment.

It is worth mentioning that in the operational deployment of BadDomains, detections generated by the system are not acted upon automatically. Instead, the system functions within a human-in-the-loop framework, where all detected domains are reviewed and verified by CERT.PL analysts before any blocking or takedown actions are initiated. This approach substantially mitigates the operational impact of false positives, as erroneous detections do not directly result in unjustified domain blocking. Prior to manual review, detected domains are automatically enriched with supporting phishing evidence by an auxiliary analysis system (referred to as phishing evidence gathering in Figure 6), which enables analysts to efficiently assess large batches of domains. While false positives may increase analyst review workload, they also provide additional signals that can reveal gaps in existing ground-truth data, including previously unreported phishing domains. Consequently, the system balances early detection benefits with operational safety, ensuring that automated detection supports, rather than replaces, expert decision-making.

Thanks to the integration of BadDomains into CERT.PL’s phishing detection environment, BadDomains’ detection results are thoroughly evaluated in a real-world scenario.

In Section 4.1, we have already presented BadDomains’ training and prediction scheme. In the deployment, we continued to retrain the model once a day and made predictions every two hours.

BadDomains deployment evaluation was performed on two periods: preliminary evaluation from 29 November 2024 to 9 April 2025 (132 days) and final evaluation from 10 April 2025 to 28 July 2025 (110 days). Unlike daily results in Section 4.6, the results here will be provided for the overall analysis period or for a moving window of 7 days because of the lower number of phishing domains than in the previous analysis (decrease from 0.85% to 0.15%). For some days, there were no phishing domains registered, which would have a strong influence on classification measures if daily calculations were kept.

The system was evaluated against Base Model (which, as already stated above, operates alongside BadDomains), and Premadoma, which was not deployed in the CERT.PL phishing detection environment, but it is still tested to give a reference. Along with the detection evaluation, we have analyzed running times of models’ training (done once per day) and prediction phases (done every 2 h). As the Base Model is a naive approach using string search mechanism, its running times can be treated as instantaneous. The other two methods mean training time was noticeably higher: For Premadoma it was 40 min, while for BadDomains it was 49 min. The prediction time for Premadoma was 15 s, which is significantly lower than for BadDomains with 22 min. BadDomains’ prediction consumed the most time on the extraction and computation of lexical, reputation, and similarity features.

Please note that during the system’s pre-deployment phase (results presented in Section 4.6), BadDomains was not part of the CERT.PL’s phishing detection pipeline and therefore had no influence on the Warning List, that is, the ground-truth of the model. Results reported for this phase rely on an independent ground truth and are free from label leakage or feedback effects. However, after deployment, our model directly affected the Warning List, which could introduce a risk of confirmation bias and label leakage. For this reason, results from the deployment phase are presented primarily to assess operational behavior and complementarity with existing detection mechanisms rather than as a fully independent estimate of classification performance.

### 5.1. Deployment Overview

The system was deployed on 29 November 2024, and the preliminary evaluation lasted from this date for 132 days up to 9 April 2025. The period covers 248,318 registered domains, from which 377 were phishing domains (0.15% of all domains).

Classification results for the preliminary deployment period are presented in Table 8 along with results for the original evaluation period of from 1 September 2023 to 31 March 2024.

The results in Table 8 show a significant drop in classification levels for two methods. $F_{1}$ dropped from 0.79 to 0.17 (62 percentage points lower) for BadDomains and from 0.42 to 0.12 for Premadoma (30 pp lower); however, an increase from 0.04 to 0.05 is seen for Base Model.

**Figure 7:**
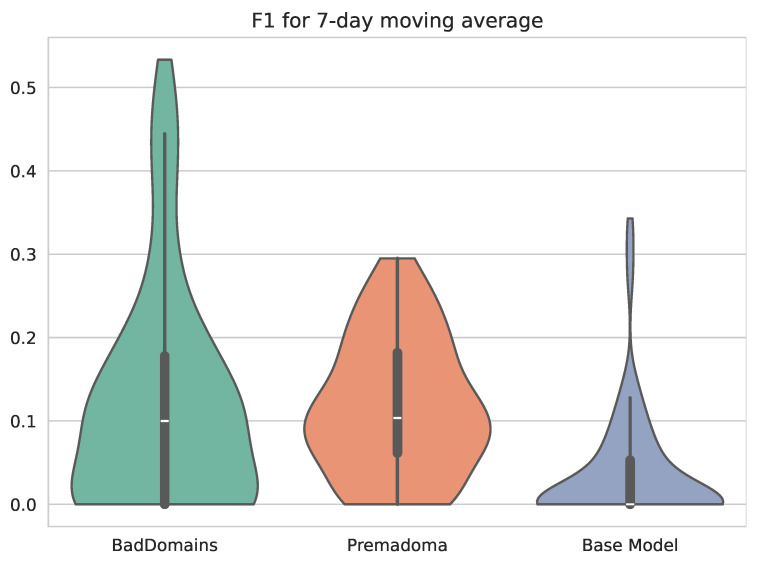
$F_{1}$ measure in the preliminary deployment period. The measure was calculated in 7-day increments.

Figure 7 presents $F_{1}$ measure in the preliminary deployment period. The measure was calculated in 7-day increments. It shows that all methods have low detection levels. BadDomains and Premadoma have a similar median value of 0.1, still higher than the Base Model. However, BadDomains’ value distribution shows more data points at levels higher than 0.3, which is also seen in higher classification levels than Premadoma in Table 8 for the analyzed preliminary deployment period.

**Figure 8:**
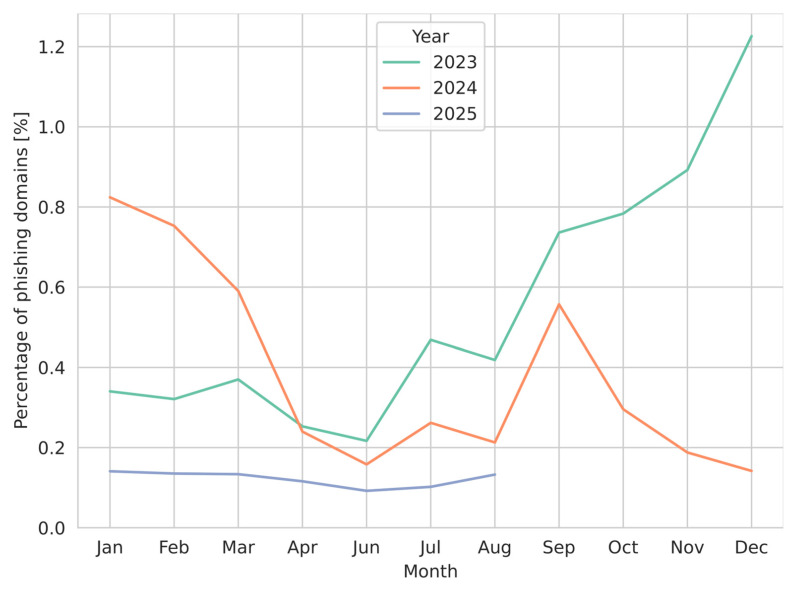
The monthly proportion of domains labeled as phishing in the ground-truth dataset relative to all newly registered domains. The figure reflects temporal variability in phishing prevalence, showing pronounced fluctuations across 2023–2025, with an overall decline in the final year and notable month-to-month variations.

As detection levels decreased for all analyzed methods, the drop can be explained by data drift observed in the source data. In the original evaluation period, 0.85% of registered domains were used for phishing purposes, while during the preliminary deployment period, the share fell to only 0.15%. Although the original dataset was imbalanced, the imbalance increased further during preliminary deployment, as the number of phishing domains decreased more than 5-fold. Figure 8 presents this decrease on a monthly scale for the years 2023–2025.

The number of phishing domains rose in the original evaluation period, achieving a maximum in December 2023 and then starting to decrease. In the preliminary deployment period, the number of phishing domains does not fluctuate as in the previous period, and is more stable, lower than 0.2%. The change in data distribution can be clearly seen here.

Data drift is understood in this paper as a shift in input data distribution. This concept is also defined by other authors as concept drift, distribution shift, dataset shift, or drift (cf. [23]). The problem of data drift is an active research area in general terms [24,25] but also in cybersecurity [26,27,28], including phishing detection [29,30,31]. In Section 5.2, we show how we addressed this problem in our work.

### 5.2. Addressing the Data Drift

As presented in Section 5.1, data drift strongly influenced BadDomains’ detection results. To address this issue, we reanalyzed data, evaluated detection results, and modified the system’s behavior.

Data reanalysis showed that cybercriminals have changed their usage behavior and do not use the *.pl* TLD to such an extent as they used to. In the original evaluation period, 14.37% of all domains on the Warning List were in the *.pl* TLD, while in the preliminary deployment period, it was 3.22%. The average daily number of domains inserted on the Warning List rose by 6.20%, with respect to the two periods. This means that cybercriminals started to use TLDs other than *.pl*. The exact reasons cannot be conclusively determined; however, other TLDs may have offered lower operational costs due to the Warning List and close cooperation between CERT.PL and *.pl* DNS registry to fight cybercrime.

Furthermore, we observed that cybercriminals more often placed phishing payloads on subdomains of registered domains. In the original evaluation period, 50.51% of the entries for *.pl* TLD on the Warning List did not have subdomains. In the preliminary deployment period, the number dropped to 28.11%. The original system’s ground-truth data was created only using effective second-level domains from the Warning List (i.e., *example.pl* or *example.com.pl*), as only those can be seen at the DNS registry level, and evidence of phishing was provided by them. With increasing usage of subdomains, we observed that some of them, impersonating well-known companies, are often used with various main domains, i.e., *company1.example1.pl*, *company1.example2.pl*. This led us to change the ground-truth creation mechanism and to add effective second-level domains for some particular cybercriminal campaigns that use the subdomain mechanism described above. This helped to extend the ground-truth set by 131 (11.33%) more domains, which helped to limit the effect of the decreased number of phishing domains in *.pl* TLD. As this change applies to both BadDomains and Premadoma—they share a ground-truth set—it was not analyzed separately, as both main models are influenced.

**Figure 9:**
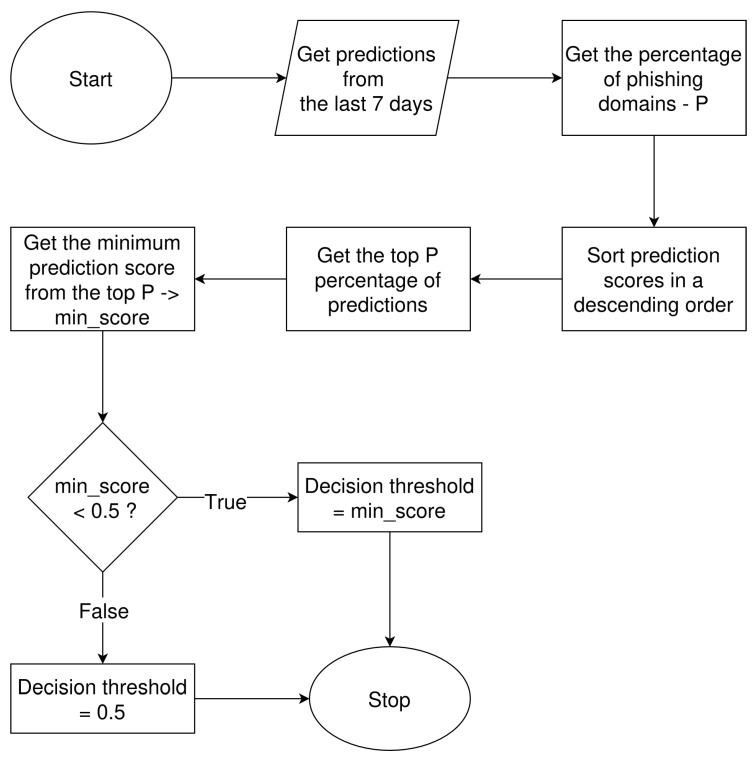
Block diagram of the decision threshold calculation mechanism.

The evaluation of detection results, as already discussed in Section 5.1, revealed a deterioration in classification performance. To address this problem, we developed a mechanism for decision threshold optimization. Adjustment of the decision threshold is a well-established approach for improving classification performance in imbalanced datasets (cf. [32,33]). Our model, like many other machine learning models, provides a prediction score for each analyzed sample. By default, the decision threshold is set to 0.5, meaning that samples with a prediction score greater than or equal to this value are classified as positive [32]. In the original evaluation period, the decision threshold remained unchanged and was set to the default value of 0.5. In the new mechanism, the decision threshold is dynamically calculated based on the historical percentage of phishing domains observed over the last 7 days. The mechanism is presented in Figure 9.

The mechanism first gets the model’s predictions from the last 7 days and calculates the percentage of phishing domains, denoted as *P*, among all those predictions. In the next step, prediction scores are sorted in descending order. Then, the top *P* percentage of predictions is obtained, and from this subset, the minimum prediction score is extracted. If this minimum prediction score is smaller than 0.5 (the default decision threshold value), then it becomes the new decision threshold value. Otherwise, the threshold remains unchanged.

The decision threshold is calculated once per day during the training phase of the model. To find the optimal number of days to consider when calculating the decision threshold, we evaluated window lengths ranging from 1 to 14 days. A 7-day period achieved nearly the best classification results when tested on the preliminary deployment period but also provided the best trade-off between stability of results (no sudden fluctuations in the number of detected domains) given by the longer periods, and speed of adjustment to changes in the dataset given by shorter periods. This value is also slightly longer than the average 5-day interval between the registration and blocking of a phishing domain, as discussed in Section 4.1, allowing the Warning List sufficient time to capture the majority of phishing domains.

### 5.3. Deployment Evaluation

After incorporating the system modifications described in Section 5.2, the final evaluation was performed on data from the period of 10 April 2025 to 28 July 2025, covering a total of 110 days. During this period, 195,570 domains were registered, of which 242 domains were identified as phishing domains (0.12% of all domains).

The classification results for the final deployment evaluation period are presented in Table 9. The results cover two versions of BadDomains—the original model without the prediction score thresholding mechanism, and the updated version that incorporates this mechanism.

Table 9 shows that BadDomains achieves the highest recall and $F_{1}$ values among all methods (0.24 and 0.30, respectively). Its recall is 12 pp higher than that of BadDomains without the thresholding mechanism, with a precision 9 pp lower, but achieving an 11 pp-higher $F_{1}$. The thresholding mechanism helped with the detection of more phishing domains (a 2-fold recall increase) at the cost of introducing 23.08% more false positive detections, which reduced precision. Nevertheless, the overall classification $F_{1}$ was improved by 57.89%. The updated version of BadDomains achieved higher $F_{1}$ levels than Premadoma and Base Model—it was higher by 21 pp and by 27 pp, respectively. Also, BadDomains outperformed both methods in terms of precision and recall.

**Figure 10:**
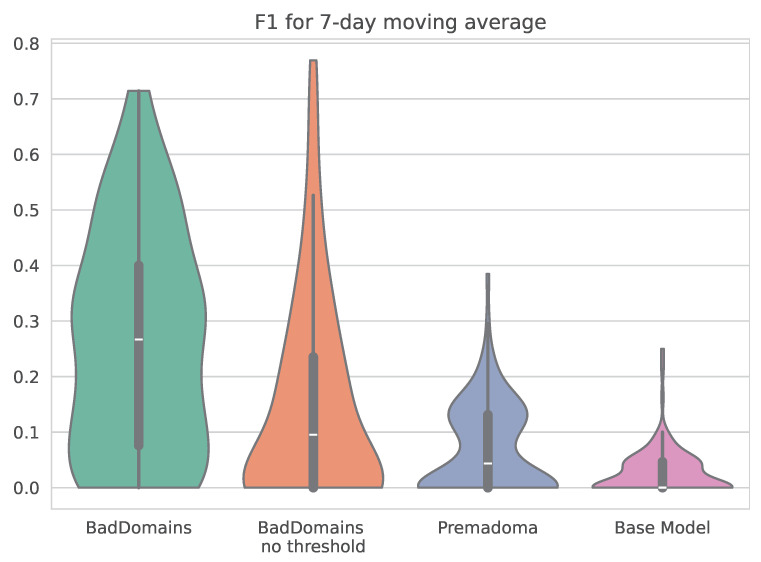
$F_{1}$ measure in the final deployment evaluation period. The measure was calculated in 7-day increments.

Figure 10 presents the $F_{1}$ score for all methods in the final deployment evaluation period, calculated in 7-day increments. The figure shows that the updated version of BadDomains achieves higher $F_{1}$ levels. Its median value is higher than those of the other methods (including the BadDomains version without a thresholding mechanism), but the value distribution also achieves higher levels—indicating that the updated BadDomains more often achieves higher values than other methods.

The analysis of the final deployment evaluation period shows that the BadDomains version with a prediction score thresholding mechanism achieves higher detection levels than other analyzed methods, including the previous version of BadDomains. Nevertheless, the achieved $F_{1}$ score is 46 percentage points lower than in the original evaluation period. For this reason, we have further analyzed detection results to better understand the system and assess whether/how it adds value to the phishing detection environment of CERT.PL.

### 5.4. Detailed Evaluation of Detection Performance

The detection results of the final deployment evaluation period have been analyzed in order to better understand the system and provide a more thorough evaluation.

In the analyzed period from 10 April 2025 to 28 July 2025, BadDomains reported 59 true positive domains. We have found that 47 (77.97%) of those domains were reported by our system before other CERT.PL monitoring systems or were reported as security incidents. Furthermore, 8 domains (13.56% of all true positives) were detected only by BadDomains and were not reported by other means. This proves that our system’s detections are faster than those from other sources of detection for the majority of cases, helping promote faster reaction to phishing activity. Its ability to detect unknown phishing domains not observed by other methods provides a means to extend detection capabilities, complementing other detection sources.

Additionally, false positive detections were analyzed further. BadDomains identified 94 domains as phishing that were absent from CERT.PL’s Warning List. These domains were analyzed using phishing website analysis services urlscan.io and VirusTotal (virustotal.com). Sixteen (17.02%) of the analyzed false positive domains showed indications of being used for phishing. For example, 11 of those domains were tagged with *dga* tag on VirusTotal, 2 were blocked on the Warning List with functional domains (e.g., *net.pl* or *com.pl*), and all those domains showed strong similarity to previous phishing domains. However, without proper URL addresses, we could not obtain a phishing payload to prove that they were, in fact, phishing. Nonetheless, those 16 domains could be treated as false negative detections in the Warning List analysis process.

The conclusions from this are two-fold. First, although the Warning List is used as the ground-truth dataset and represents a curated and highly reliable source, it has inherent limitations. Domains are added to the Warning List only after manual verification by human analysts based on proof that a phishing payload was present on the domain. Additionally, cybercriminals employ cloaking techniques to hide phishing payloads from the analysts (cf. [34]). That is why some of the domains could be missed by the Warning List, as there was no proof of phishing activity, or domains were not reported by any source.

Second, if those 16 false negative domains were treated as true positives from BadDomains, then the $F_{1}$ score would increase by 22 percentage points. This suggests that examining domains flagged as false positives by BadDomains may yield valuable insights into uncovering false negatives in the Warning List itself.

## 6. Conclusions and Future Work

In this paper, we have presented BadDomains, a system for the early detection of phishing registrations in the *.pl* TLD. We have compared it to a similar system created for the *.eu* TLD called Premadoma and a baseline method. Our classifier, based on LightGBM, was trained on features originating from the literature as well as from our own research. It demonstrated a high $F_{1}$ score of 0.79, significantly outperforming Premadoma by 37 percentage points and the Base Model by 75 percentage points.

BadDomains was successfully deployed as a part of CERT.PL’s phishing detection environment, giving an opportunity to evaluate the system in a real-world, operational scenario. From an operational perspective, BadDomains is designed to support analysts rather than act autonomously. All detections are reviewed by CERT.PL analysts before any blocking actions are taken, which limits the practical impact of false positives and prevents unjustified domain mitigation. While false positives may increase review effort, this human-in-the-loop design ensures safe deployment and allows the system to complement existing detection workflows without introducing undue operational risk. During the preliminary deployment, the classification results showed lower levels of detection for all models analyzed—the BadDomains $F_{1}$ score was lower by 62 percentage points than during the original evaluation period. The classification level deterioration was caused by a data drift: The number of phishing domains decreased from 0.85% of all domain registrations to 0.15%. We addressed this issue by developing a decision threshold mechanism, which helped to achieve an 11 percentage points higher level of $F_{1}$ than the system’s version without this mechanism.

In the preliminary deployment period, Premadoma achieved a 5 percentage point-lower $F_{1}$ value than BadDomains, with 11 percentage points higher recall, but similar 7-day results. Introduction of the thresholding mechanism in BadDomains helped to achieve an 11 percentage points higher $F_{1}$ value than in the model without this mechanism—including a 12 percentage points higher recall level.

The Base Model performed worse than the other two methods in all evaluation periods. Utilization of only keywords’ presence for detection proved to be insufficient. Such a detection strategy is highly dependent on the keyword list being up-to-date, as cybercriminals tend to change words used in registered phishing domains. As our analysis showed, only using keywords along with other features, as in BadDomains, can help achieve better classification results.

Despite the lower detection levels of all analyzed methods during the deployment period than during the original evaluation, we proved that BadDomains achieved better classification results than the other two methods, including Premadoma. The results showed that levels of both precision and recall were higher in general for BadDomains, but also when averaged by days/weeks. When considering the running times of the methods, it can be argued that the higher detection performance of BadDomains compared to the other two methods comes at the cost of higher resource usage. While the training time of Premadoma and BadDomains is comparable, the prediction time for BadDomains is significantly longer. We believe this trade-off is acceptable, as the prediction time does not overlap with subsequent prediction batches, and the hardware requirements are not high compared to those of other machine learning architectures, e.g., Large Language Models [35]. Moreover, with code optimization and improved hardware, the prediction time could be further reduced.

BadDomains’ deployment phase results are hard to compare to other real-world-operating phishing domain detection systems based on the registry data. First, we are not aware of any direct equivalents to our system. Public systems such as Premadoma (*.eu* TLD), Domain Watch (*.uk* TLD [36]), RegCheck (*.be* TLD [17]), and READS (*.it* TLD [18]) focus on the detection of malicious domains or anomalous domain registrations, while our system detects phishing domain registrations, and, as such, those objectives do not overlap. Second, authors of the systems mentioned above present results matching their research objectives, which do not fit ours, and they report measures on different entities, for example, malicious domains or anomalous registrations. The provided detection results show information about precision, recall, or F1, but sometimes even that information is limited. For example, Premadoma’s prediction results are presented for the validation phase, while for the deployment phase, the authors report only the number of blacklisted domains. Domain Watch’s public information about detection results shows 60% precision [37] but without information about the deployment phase or any other evaluation information. RegCheck’s performance for the *.be* TLD in the deployment phase omits information about typical binary classification measures (precision, recall, F1) [17], and for historical data for the *.nl* TLD reported performance is 22.08% for precision and 47.80% for recall. These results show similar values to BadDomains’ deployment phase but also show how complex and non-trivial this detection problem is. To perform a fair comparison of the systems, detection analysis should be performed using data from other TLD registers. However, this approach is practically impossible due to the usage of Personally Identifiable Information by BadDomains, which would be a legally difficult task if transferred outside the original registry. Despite the fact that BadDomains’ detection results for deployment phase are lower than for the pre-deployment phase, we decided to publish these results to show how detection efficiency can deteriorate due to external factors and what can be done to fight such an effect, but also to show that with even less favorable detection levels, our deployed system provides early detection, effectively extending detection coverage.

A detailed analysis of detection results during the deployment period showed that nearly 78% of domains were reported first by BadDomains, demonstrating that it consistently detects threats earlier than other mechanisms operating in the CERT.PL phishing detection environment. In contrast, as illustrated in Figure 3, existing detection processes without BadDomains typically identify approximately 80% of phishing domains within five days after registration, with some cases being delayed by up to 80 days. In addition, 14% of domains were detected only by BadDomains, indicating that the system provides novel information not available from other detection sources and effectively extends overall detection coverage. Furthermore, analysis of BadDomains’ False Positive results showed potential for improving the ground-truth data coverage by uncovering its False Negatives.

For the above reasons, we believe that BadDomains proved its usefulness as an early detector of phishing domain registrations, offering a reliable tool for blocking cyberattacks. It extends other methods of phishing detection in CERT.PL’s environment, helping to improve the safety of the *.pl* TLD and internet users.

One of the primary challenges in developing this system was its focus on early detection, which limited the number of variables available for use. For instance, the target website may not yet exist, complicating the use of variables related to the website’s content. Additionally, the information is provided by registrants and can contain both intentional and accidental errors. A natural continuation of the project is to implement an additional stage in which, upon the creation of a website, further analysis of the URLs can be conducted. Another significant challenge we faced was the incomplete list of ground truth, as it often takes several days for the data to become fully annotated. To overcome this, we introduced a five-day interval between the training and testing datasets, with a mechanism to include any phishing observations that occur during this period into the training set. This approach helps the system quickly adapt to the evolving tactics of cybercriminals.

The proposed method utilizes Personally Identifiable Information (PII) stored in the *.pl* TLD registry. We have performed legal analysis of PII usage in our method with our internal data protection officer and legal team. We have also analyzed the research ethics of this usage and concluded that the outcomes of our work will have a positive impact on the security of the *.pl* TLD and will help with the protection of Polish internet users against phishing. In our opinion, the usage of PII in our method is necessary, as from our observations, cybercriminals use not only random, fake data, but also try to imitate information provided by real entities. Without direct analysis of the PII, our method potentially would not be as effective as it is. We put much effort into the mitigation of the potential security risks introduced by the usage of PII. The analyzed data never left our internal network, and all data enrichment services were deployed internally. All analysts and researchers involved in the work on our method are employees of the *.pl* TLD registry, are bound with a non-disclosure agreement, and must comply with strict internal data processing and security procedures. Additionally, the registry data were normalized and sanitized before analysis in order to prevent any models’ software manipulation.

Although Premadoma was adapted for the purposes of this study, its precision and recall remained low in the evaluated setting. This outcome highlights a limitation of this work. The observed performance may be influenced by structural differences between the *.pl* TLD and the environments for which Premadoma was originally designed as well as by differences in ground-truth definition, which was broader in the case of Premadoma. Nevertheless, Premadoma was included to provide a reference baseline, as no alternative publicly available models suitable for early phishing domain detection in this operational context were available for comparison.

While our experimental evaluation focuses exclusively on the *.pl* TLD, we emphasize that this choice was motivated by the availability of high-quality, operational registry data and the ability to validate the system in a real-world CERT environment. The *.pl* namespace represents a mature TLD with heterogeneous registrants and attack patterns, making it a well-defined and realistic case study for early phishing detection at registration time. Importantly, the core components of BadDomains—including the LightGBM-based classifier, the feature engineering methodology, the early-detection paradigm, and the decision-threshold mechanism designed to mitigate data drift—are inherently TLD agnostic. At the same time, we acknowledge that generalization to other TLDs may be influenced by factors such as differences in registry data schemas, availability and legal permissibility of registrant metadata, and TLD-specific registration policies. Future work will therefore focus on validating the approach on additional TLDs as well as identifying minimal feature subsets that preserve performance under more restrictive data-access conditions.

We are aware that the proposed systems require some improvements. In the presented work, we have focused on practical methods to address the effects of external factors negatively impacting detection effectiveness. A detailed feature-level analysis could uncover more information regarding the model and influence of features on detection as well as its reliance on *.pl* TLD-specific data patterns. Additional work could be done to extend the feature set, for example, by applying text embedding methods to textual fields. Information about the domain’s life-cycle could also be incorporated into the detection method. Also, some other registry data verification systems could be adapted to phishing detection to provide a baseline for our method. Furthermore, methods for better integration with phishing payload evidence gathering systems could be improved. This could help with uncovering even more phishing domains by extending the ground-truth dataset. All these suggestions should be considered for future work.

## Figures and Tables

**Table 1:** Overview of novel or partly novel features used in modeling with corresponding descriptions.

Feature Name	Description	Feature Group	Novel?
Address is valid	Level of the address validity according to Nominatim.	Contact	Partly
Phone number length	The length of a client’s phone number.	Contact	Yes
Phone is valid	Indicator of whether the phone number is syntactically valid.	Contact	Yes
Phone’s country	Client’s origin country based on phone number.	Contact	Yes
{F} is country consistent	Indicator of whether the country code provided in client information is consistent with data in the field F = {address, phone}	Contact	Partly
Name anonymized	Indicator of whether the name was anonymized using privacy services.	Contact	Yes
Sponsor is creator	Indicator of whether the last register was the one creating the domain	Contact	Yes
Creator is client information creator	Indicator of whether the last register updating the client info was the one creating the domain	Contact	Yes
Number of {F} per client	Number of phone numbers or emails per client. F = {phone, email}	Contact	Yes
Postcode consistency	Indicator whether the postcode is consistent with data from Nominatim. Different values depending on the level of consistency or problems.	Contact	Partly
Client name in email	Indicator of whether registrant name occurs in email address	Contact	Partly
Number of occurrences of {F} in {T} days	Number of occurrences of F = {email, phone, client name} in the previous T = {14, 30} days.	Contact	Partly
{F} is blank	Indicator of whether a value of the field F is blank F = {client postal code phone number, email, city, name, country code, nameserver, nameserver IP}	Contact	Yes
Phone number is suspicious	Indicator of whether the phone number is on the curated list of suspicious numbers.	Reputation	Yes
{F} on the blacklist	Indicator of whether F = {email, phone} is on the blacklist of contact information used for phishing.	Reputation	Partly
Keywords in domain	Indicator of whether a keyword from the incident-handlers curated keyword list is in the domain.	Keywords	Yes
{M} distance {D} to keywords	M = {mean, min, max, median} measure of the distance metric D = {Damerau, Jaro}, where the domain name is split into smaller tokens. For each token, the maximum similarity score with elements from the incident-handlers curated keyword list is computed.	Keywords	Yes
{F} has 3 consecutive chars	Indicates of whether the provided entry in field F = {email, city, street} contains three consecutive identical characters or numbers.	Lexical	Yes
Number of Polish chars in F	Number of Polish characters in field F = {client name, city, street}	Lexical	Yes
Percentage of Polish chars in F	Percentage of Polish characters in field F = {client name, city, street}.	Lexical	Yes
Phishing in 90D in {F}	Indication of whether there were any phishing domains in the past 90 days for field F = {email, phone}	Reputation	Yes
Maximum Levenshtein distance to previous {N} phishing domains	Maximum Levenshtein distance similarity score between the domain name and the names of the last N = {10, 50, 100} phishing domains in the dataset, arranged in chronological order.	Similarity	Partly
{M} Levenshtein distance to previous {N} domains	{Maximum, mean} Levenshtein distance similarity score between the domain name and the names of the last N = {10, 50, 100} domains in the dataset, arranged in chronological order	Similarity	Partly
Maximum similarity score {D} with top-affected targets	Maximum D = {Levenshtein, Jaro} similarity score with the most-affected impersonation targets.	Similarity	Yes
Contains top-affected target	Indicator of whether domain name contains a name from the list of the most-affected impersonation targets.	Similarity	Yes
Seconds to next registration	Time in seconds to registration of the next domain	Time	Yes
Closest domain creation time difference	Time in seconds between registration of the next and previous domains	Time	Yes
Domain to client creation time	Time between the creation of the client information and the registration of the domain	Time	Yes
Number of domains in the last {T}	Number of registered domains in the preceding T = {30 s, 1 min, 5 min, 10 min}	Time	Yes

**Table 2:** Overview of non-novel features used by BadDomains. Features in the Lexical group use information from domain name, client’s name, client’s city, and client’s street registry fields.

Feature Name	Description	Feature Group
Creation timestamp	Creation timestamp	Time
Creation hour	Creation hour	Time
Creation day	Creation day	Time
Registration period in months	Registration period in months	Time
Number of seconds since last registration	Number of seconds since last registration.	Time
Reputation score in the last {T} days for field {F}	Reputation score in the last T = {14, 30, 90} days for field F = {domain registrant, domain updating entity, nameserver, client’s email provider, creator of a client record, updater of a client record, creator of a nameserver record, updater of a nameserver record}.	Reputation
String length	Length of the string in the field.	Lexical
Words number	Number of words in the field.	Lexical
Mean length of words	Mean word length in the field.	Lexical
Entropy	Character entropy in the field.	Lexical
Number of unique characters	Number of unique characters in the field.	Lexical
Unique characters percentage	Percentage of unique characters in the field.	Lexical
Maximum number length	Maximum length of a number string in the field.	Lexical
Number of digits	Number of digits in the field.	Lexical
String contains a digit	A digit is present in the field.	Lexical
String offset of a maximum number	String offset of the string with maximum number in the field.	Lexical
Relative string offset of a maximum number	String offset of the string with maximum number in the field, relative to the string length.	Lexical
Percentage of digits	Percentage of digits as a part of the string in the field.	Lexical
Number of letters	Number of letters in the field.	Lexical
Percentage of letters	Percentage of letters as a part of the full string in the field.	Lexical
Number of non-Latin characters	Number of non-Latin characters in the field.	Lexical
Number of special characters	Number of special characters in the field.	Lexical
Number of dashes	Number of dashes in the field.	Lexical
String contains a dash	A dash is present in the field.	Lexical
String contains a dot	A dot is present in the field.	Lexical
Number of vowels	Number of vowels in the field.	Lexical
Percentage of vowels	Percentage of vowels as a part of the full string in the field.	Lexical
Number of consonants	Number of consonants in the field.	Lexical
Percentage of consonants	Percentage of consonants as a part of the full string in the field.	Lexical
Client’s email length	Client’s email length.	Lexical
Number of unique characters in client’s email	Number of unique characters in client’s email	Lexical
Number of digits in a client’s email	Number of digits in a client’s email.	Lexical
A digit is present in a client’s email	A digit is present in a client’s email.	Lexical
Number of dashes in a client’s email	Number of dashes in a client’s email.	Lexical
A dash is present in a client’s email	A dash is present in a client’s email.	Lexical
Number of dots in a client’s email	Number of dots in a client’s email.	Lexical
A dot is present in a client’s email	A dot is present in a client’s email.	Lexical
Nameserver has an IP address record	Nameserver has an IP address record.	Nameserver
Nameserver’s company	Nameserver’s company	Nameserver
Nameserver’s location	Nameserver’s location	Nameserver
Autonomous System Number	Autonomous System Number	Nameserver
Client’s country from Nominatim	Client’s country from Nominatim	Contact
Client’s e-mail provider	Client’s e-mail provider	Contact
Domain registrant	Domain registrant	Basic
Domain updating entity	Domain updating entity	Basic
Domain zone	Domain zone	Basic
Client’s country	Client’s country	Basic
Client is an organization	Client is an organization.	Basic
Creator of a client record	Creator of a client record	Basic
Updater of a client record	Updater of a client record	Basic
Creator of a nameserver record	Creator of a nameserver record	Basic
Updater of a nameserver record	Updater of a nameserver record	Basic
Nameserver’s IP version	Nameserver’s IP version	Basic

**Table 3:** Data split.

Name	Start Date	End Date	Number of Batches	Number of All Domains	Number of Phishing Domains [Percentage of All Domains]
valid	1 July 2023	31 August 2023	8 weeks/62 days	114,899	613 [0.53%]
test	1 September 2023	31 March 2024	213 days	460,636	3950 [0.85%]

**Table 4:** The main differences between the Premadoma and BadDomains methods.

Property	Premadoma	BadDomains
**ML architecture**	Set of two binary supervised classifiers and one that is clustering based, integrated into majority voting classifier	Supervised binary classification
**ML algorithms used**	PART algorithm (hierarchical shrinkage algorithm in our deployment) + agglomerative clustering algorithm	LightGBM
**Detection focus**	Malicious campaign domain detection and grouping	Integration of operational knowledge into detection process
**Feature types**	Classification performed mainly on reputation-based and simple lexical features; clustering mainly based on similarity of registrant information. Single features based on time and name server data.	Eight feature groups, with novel features in registrant validation-, keyword-, reputation-, time-, and similarity-based groups.
**Operational intelligence usage**	Limited to usage of domain name blacklist	Domain name blacklist, keywords of particular interest, current top-affected targets, additional incident handler-curated list of suspicious phone numbers
**Domain name similarity features**	Limited to similarity with blacklisted domain names	Similarity to previous phishing domain names, top-affected targets, keywords of particular interest
**Data validation and consistency features**	Limited to registrant’s address validity score	Registrant address validity, phone number syntactic validation, checking if any information is missing, consistency of country code and postcode.

**Table 5:** Hyperparameter search space and optimal values of BadDomains by maximizing $F_{1}$.

Name	Search Space	Optimal Value (Max $F_{1}$)
n_days_train	[7, 14, 30, 60, 90]	90
add_phishing_history	[False, True]	True
undersampling	[False, True]	False
sampling_strategy	Uniform [1, 100]	-
max_depth	[3, 4, 5, 6, 7, 8]	6
n_estimators	RandInt [100, 5000]	2274
learning_rate	LogUniform [0.0001, 0.2]	0.017
colsample_bytree	Uniform [0.4, 0.9]	0.891
reg_alpha	Uniform [0.0, 1.0]	0.554
min_child_samples	RandInt [5, 200]	163
is_unbalance	[False, True]	False

**Table 6:** Possible values of hyperparameters for models used in our adaptation of Premadoma and optimal values.

Name	Search Space	Classifier 1	Classifier 2	Clustering
Distribution spread	range (0.2, 1, 0.05) or $2^{i}\times 10^{-5}$, i∈ {0, 1, …, 9} or $2^{i}$ × 0.01, i∈ {0, 1, …, 4}	0.35	0.00512	-
Blacklist incompleteness	range (0.55, 1.0, 0.05)	0.55	0.55	-
Training window	[15, 30, 45, 60]	60	15	15
Distance threshold	range (0.5, 1.0, 0.05)	-	-	0.55
Minimum cluster size	range (5, 50, 50)	-	-	15

**Table 7:** Average daily performance metrics with standard deviation across the proposed model (BadDomains), established benchmark (Premadoma), and simple Base Model on test data (September 2023–April 2024). The best results are highlighted in bold.

Model	Precision	Recall	$F_{1}$
Premadoma	0.32 ± 0.18	0.56 ± 0.30	0.39 ± 0.21
Base Model	0.02 ± 0.02	0.36 ± 0.22	0.04 ± 0.03
BadDomains	**0.73 ± 0.20**	**0.83 ± 0.16**	**0.76 ± 0.17**

**Table 8:** Classification results for the original evaluation period from 1 September 2023 to 31 March 2024 and preliminary deployment period from 29 November 2024 to 9 April 2025. The best results are highlighted in bold.

Analysis Period	Method	Precision	Recall	$F_{1}$
Original evaluation	BadDomains	**0.74**	**0.83**	**0.79**
	Premadoma	0.33	0.58	0.42
	Base Model	0.02	0.36	0.04
Preliminary deployment	BadDomains	**0.38**	0.11	**0.17**
	Premadoma	0.09	**0.22**	0.12
	Base Model	0.04	0.07	0.05

**Table 9:** Classification results for the deployment period from 10 April 2025 to 28 July 2025, after integration of the prediction score thresholding mechanism. The best results are highlighted in bold.

Method	Precision	Recall	$F_{1}$
BadDomains	0.39	**0.24**	**0.30**
BadDomains no thresholding mechanism	**0.48**	0.12	0.19
Premadoma	0.08	0.12	0.09
Base Model	0.02	0.06	0.03

## Data Availability

The dataset presented in this article is not available for legal reasons as it contains Personally Identifiable Information.
